# Should We All be Scientists? Re-thinking Laboratory Research as a Calling

**DOI:** 10.1007/s11948-017-9940-0

**Published:** 2017-07-19

**Authors:** Louise Bezuidenhout, Nathaniel A. Warne

**Affiliations:** 1 Institute for Science, Innovation and Society, University of Oxford, 64 Banbury Road, Oxford, OX2 6PN UK; 2 Steve Biko Centre for Bioethics, Faculty of Health Sciences, University of the Witwatersrand, Johannesburg, South Africa; 3 Center for Theology, Science and Human Flourishing, University of Notre Dame, Notre Dame, IN 46556 USA

**Keywords:** Science practice, Virtue ethics, Calling, Responsible conduct of research, Bioethics, Conduct

## Abstract

In recent years there have been major shifts in how the role of science—and scientists—are understood. The critical examination of scientific expertise within the field of Science and Technology Studies (STS) are increasingly eroding notions of the “otherness” of scientists. It would seem to suggest that anyone can be a scientist—when provided with the appropriate training and access to data. In contrast, however, ethnographic evidence from the scientific community tells a different story. Scientists are quick to recognize that not everyone can—or should—be a scientist. Appealing to notions such as “good hands” or “gut feelings”, scientists narrate a distinction between good and bad scientists that cannot be reduced to education, access, or opportunity. The key to good science requires scientists to express an intuitive feeling for their discipline, but also that individuals derive considerable personal satisfaction from their work. Discussing this personal joy in—and “fittingness” of—scientific occupations using the fields of STS, ethics and science policy is highly problematic. In this paper we turn to theology discourse to analyze the notion of “callings” as a means of understanding this issue. Callings highlight the identification and examination of individual talents to determine fit occupations for specific persons. Framing science as a calling represents a novel view of research that places the talents and dispositions of individuals and their relationship to the community at the center of flourishing practices.

## Is Everyone a Scientist Now?

As scientific research has developed over the last century, so has the manner in which scientists are viewed by society. Science and technology scholars will be familiar with the three-wave model that details the changing attitudes to science over the course of the last century (Collins 2014: 21). In the early part of the twentieth century the role of “history, philosophy and sociology of science was clear: explain how the scientific miracle worked” (Collins 2014: 21). In the 1960s Thomas Kuhn’s book, *The Structure of Scientific Revolutions* (1962) changed the manner in which the scientific method was understood, highlighting the irregularity of “paradigm shifts” in scientific research. This was accompanied by the rise in the 1970s of the field of the ‘sociology of scientific knowledge” that suggested that “the social explanations of what are counted as true scientific facts and findings should be of the same sort as were used to explain false scientific facts and findings (Collins 2014: 28). The third wave in the field of science studies has tended to focus on issues of expertise and experience—such as Harry Collins’s work on tacit knowledge. These studies tend to critically interrogate the boundaries between those termed “experts” and the public (Collins and Pinch 1993; Collins 2014).

This latest trend in science studies has drawn considerable attention to the possibility that scientists are distinguished from the public only by their acquisition of certain types of expertise. In his book, *Are We All Scientific Experts Now?* Collins delineates three different types of expertise: ubiquitous, specialist, meta, and default (Collins 2014: 115–131). While Collins agrees that not everyone is a “scientific expert”, he suggests that “without changing society, small numbers of initially ordinary people can become scientific experts, not through reading but through specialist experiences at work, or experience of chronic diseases, or extended discourse with existing experts (Collins 2014: 131–132). In his book, Collins draws attention to the fact that “[w]e are not all *meta*-*experts* when it comes to judging technical matters … And we are not all *default experts* because we do not share the scientific ethos which may be the most valuable contribution of science to society. He argues that science needs to be re-elevated to a “special position in our society” (Collins 2014: 132).

The work of Collins and his peers emphasizes the drift in public understanding of science away from the first-wave notion of science as an untouchable province of expertise accessible to few. This “popularization” of science has been mirrored by the recent explosion of public engagements in science. Moreover, the impression that science can be done with the right training, access to data and equipment, and motivation is also echoed in the DIYBio movement. The emergence of “citizen scientist” communities in the early part of the twenty-first century has challenged any remaining preconceptions of science as the province of an elite. Indeed, a number of papers have suggested that the emergence of these citizen science groups can be understood as a “de-skilling” of science (Calvert 2013; Evans and Selgelid 2015)—suggesting that the barrier between scientist and non-scientist is a matter of interest and exposure to resources rather than any other measure.

Interestingly, these echoes of inclusion are also present in changes in modern funding for scientific research. Governments around the world are making science, technology and innovation (STI) funding a priority and actively recruiting young people into science, technology, engineering and mathematics (STEM) subjects, and making research and development (R&D) a key element of their long-term investments. This commitment is reflected in a 2015 speech by US president Barack Obama, where he said:

Part of what’s important to do is also to recognize that what you do in math and engineering and science has a purpose to it; that there are huge challenges that we have to solve in how we have clean energy, and how to we clean up our environment, and how do we solve crippling diseases like Parkinson’s or Alzheimer’s. And when we give students the inspiration not just that math and science are inherently interesting, and technology and engineering are inherently interesting, but there’s actual problems to solve, it turns out that young people, they rise to the challenge. And that’s what’s so exciting about it.

We don’t want to just increase the number of American students in STEM. We want to make sure everybody is involved. We want to increase the diversity of STEM programs, as well. And that’s been a theme of this science fair. We get the most out of all our nation’s talent – and that means reaching out to boys and girls, men and women of all races and all backgrounds. Science is for all of us. And we want our classrooms and labs and workplaces and media to reflect that …..

So it’s not enough for us to just lift up young people and say, great job, way to go. You also have to have labs to go to, and you’ve got to be able to support yourself while you’re doing this amazing research. And that involves us as a society making the kind of investments that are going to be necessary for us to continue to innovate for many, many years to come.^1^

The drive towards attracting and retaining individuals within scientific research is also reflected in the increasing attention being paid to science pedagogy. Increasingly advanced discussions about science pedagogy undoubtedly focus on *keeping students in science.* This, it is important to recognize, is not only by stimulating interest in science, but by facilitating the acquisition of science knowledge and skills (see Henriksen et al. 2015 for examples). Indeed, campaigns such as “Educate to Innovate” in the USA directly engage with rolling out and improving science education, so that *“no young person in America should miss out on the chance to excel in these fields just because they don’t have the resources”.* 2

Together, these trends seem to suggest that anyone can be a scientist. Indeed, current rhetoric—together with the emergence of community scientists such as the DIYBio movement—suggest that the number of individuals conducting scientific research is limited solely by opportunity. The current climate thus focuses on increasing the opportunities to get involved in science—with the underlying assumption that anyone availing themselves of these opportunities has in themselves the potential to be effective scientists. While such rhetoric is inspiring, it is also possible that it is misleading, as it overlooks key issues such as individual talent, enjoyment and intuition. Are some individuals—regardless of opportunity—not suited to careers in science?

## The Value of “Good Pair of Hands”

Between August 2016 and February 2017 one of the authors (LB) was embedded within two molecular biology laboratories in the USA.^3^ During this period the author conducted laboratory research, and engaged with scientists within the laboratory. A key element of this ethnographic research was understanding how skill acquisition occurred through daily research practices. Unsurprisingly, the focus of this research lead to considerable discussions about *what made a good scientist.*

Amongst most of the participants in both the laboratories visited, the notion of “good hands” came up regularly in conversation. Participants made comments such as: *“He has good hands”* [US1/18/10/16], or conversely: *“She doesn’t just have bad hands, she doesn’t know what she’s doing”* [US2/19/10/16]. What the participants were highlighting was not necessarily a learnt tacit ability amongst their peers, but rather what they perceived to be an innate ability and affinity that some individuals had towards their laboratory work. When pressed on this issue, most participants agreed that you just couldn’t “teach a good pair of hands”.

Similarly, when discussing their research, many of the participants referenced a deep sense of joy and fulfillment that came out of their laboratory work. One participant, for example, when talking about the bacterial strains he was culturing, made reference to them as: *“my babies”* [US1/18/10/16]. When discussing them, or showing off their growth to the laboratory he got very excited and quite emotional. Another participant in the same laboratory, when talking about her peers and their enthusiasm, said: *“If we’re spending this much time in the lab, we might as well be excited about what we’re doing!”* [US1/19/09/16].

These comments are particularly revealing, as they highlight a key distinction that is largely overlooked in discussions on science: that not everyone is equally suited to a career in science (particularly bench science). As evident in the comment above (*“She doesn’t just have bad hands, she doesn’t know what she’s doing”* [US2/19/10/16]), scientists working at the bench often made a distinction between what can be taught, and the innate talents that make for excellence in science.

While none of the participants suggested that any of their colleagues were less than competent in their work, their comments highlighted a distinction made within the scientific community between individuals who were ultimately suited to careers in science and those who—for whatever reason—were not. Such observations correlate with other ethnographic studies on scientists and scientific research practices. Authors such as Latour and Woolgar (1979) and Traweek (1992) have spent extended periods of time within scientific laboratories investigating how scientists conduct daily research, and how they discuss their own work—and that of their colleagues. Examining these texts provides key insights not only into how scientists discuss their research, but how they discuss themselves within the research process. Together, all these studies must draw our attention to something that is recognized by scientists: that within a cohort of competent scientists some are more suited to careers in science than others.

## Confronting the Impasse

If scientists recognize such distinctions—and talk about them in the course of their daily research—it would thus seem important that such issues are addressed in commentaries of science. Nonetheless, raising such discussions within STS, life science ethics and science policy remains highly challenging. As detailed above, the acquisition-of-expertise focus within current STS, as well as the drive towards educating scientists inherent within life science ethics and policy, all create a straw man guarding discussions rather than examining the issue in depth.

While disciplines such as psychology offer ways to discuss *aptitude*—or natural ability—for science, it nonetheless remains an insufficient concept. Indeed, two key elements continue to be overlooked—the *tacit ability* necessary in scientific research, and the need for joy, curiosity and passion. While many individuals may show above normal aptitude for scientific disciplines, without these two elements it is unlikely that they will flourish within a laboratory setting. By consequence, the key distinction between proficiency and excellence continues to be largely overlooked. Thus, we must ask, what can be done to open up the ethics discourse to enable the proficiency/excellence distinction to be discussed without unintentionally creating distinctions that may prejudice opinion against science practitioners not demonstrating excellence.

## Reframing Science as a Calling

A new, person-centric and contextual model of scientific practice would thus seem necessary to breathe new life into discussions on the recruitment and training of new scientists—particularly one that distinguishes between proficiency and excellence. Although often overlooked for its theological heritage, the literature of professions as “callings” has the potential to contribute to the problems identified above. At least minimally, the model of a career as a calling draws attention to the natural inclination towards certain talents that predisposes individuals for certain activities over others. Thus, the idea of science as a “calling” raises the possibility that a specific combination of inclination, talent and support are necessary to be an effective scientist. Moreover, it suggests that not all individuals—and indeed not all scientists—are equally suited to be scientific researchers.

### What is a “Calling”?

William Perkins, a sixteenth century Protestant theologian, defined a calling as: “that … which belongs to some particular men: as the calling of a magistrate, the calling of a Minister, the calling of a master, of a father, of a child, of a servant, of a subject, or any other calling that is common to all” (Perkins 1603: 13).^4^ Perkins writes, “every man must judge that particular calling, in which God hath placed him, *to be best of all calling for him: I say not simply best, but best for him*” (Perkins 1603, *emphasis added*). The calling of any person can be understood as the ideal occupation—or vocation—based on their individual and unique being.

One need not be concerned with the theological language here. What Perkins is describing is that each person has particular callings that relate both to their natural gifting and talents as well as their immediate social surroundings. Indeed, recent work in the field of psychology that uses the concept of callings in the consideration of vocation that has no obvious religious ties, showing the strength of the concept beyond theology. One example of this is Douglas Hall and Dawn Chandler’s use of the concept of calling in their own research on purpose in psychological success and careers (Hall and Chandler 2005). Indeed, psychologists recognize that the word talent, which is still significantly drawn upon in secular cultural speech, has biblical and theological roots (Csikszentmihalyi et al. 1993: 21).

Callings are by no means exclusive, and one can hold more than one calling simultaneously. For example, one can be both called to be a scientist and a mother simultaneously. Where one calling is related to natural character traits and dispositions toward one activity or another—say the kind of detail orientedness necessary for the practice of science or the keen ear that allows one to learn a musical instrument with ease—the other calling could be related more to immediate surroundings, such as the calling to be a mother, or neighbour.

As callings are by no means exclusive, it is also of importance to recognize that the different callings held by one individual may at times compete for time and resources. The expectations relating to the calling of being a mother, for example, may be in direct competition with being an effective scientist. In such situations the doctrine of calling emphasizes the role of the community in helping people realize appropriate boundaries and how to navigate said boundaries. Indeed, the community plays a vital role in assisting the individual in not only mediating the demands of their individual callings, but also critically evaluating the callings to which s/he has pledged allegiance.

Key points to consider from science as a calling thus include:

- Calling is a particularization of virtue, so its character is dictated by what it is to be a particular, properly functioning human, and that is not going to be the same across the board.^5^
- While callings are a universal moral good, they also take the character of calling to be indexical, meaning that its intention (in particular instances) is contextually sensitive and is determined by the character of the person in whom it is instantiated, or have a specific instance in a particular context. Thus, a combination of inherent talents, circumstances, and relations with other people are necessary for individuals to be called to the practice of science.
- That calling involves the particularization of virtues, and is thus a moral duty.
- That the recognition of callings is something that an individual must discover for themselves, while being assisted in reflection by their peers.

### Flourishing in a Calling

An individual in the right calling has the opportunity to “flourish”, a term consistent with what ancients like Plato and Aristotle called *eudaimon*ia, which is the best life for humans, a life of happiness and flourishing. The notion of “flourishing” also links to contemporary psychology (For example, Csikszentmihalyi 1990). The theological tradition in which we are drawing has much to say about what it means to flourish. Just as there is a sense in which calling can be taken in the two senses mentioned above, the spiritual^6^ and vocational, flourishing also has a twofold sense. The latter is penultimate and can be experienced in this life under the right conditions, and could be considered a kind of “natural” flourishing.^7^ It is important to note that even within the theological tradition, those without religious faith who find enjoyment and fulfillment in their work and family, can experience natural flourishing (See Biggar 2006). Indeed, the development of character identifies a goal of flourishing (consciously or unconsciously) and habituates virtues and practices to achieve this end.^8^ What we mean by flourishing in the specific case of calling and work is a concurrence of one’s activates with one’s talents, their innate loves with their occupations. It is through these activities and the development of virtues that one can have a sense of flourishing.

We here take virtue in an Aristotelian-Thomist sense, meaning a virtue is a disposition to do the right thing and to have the right feelings and emotions in various areas of life. Aristotle separates virtue into two kinds: intellectual and moral (Aristotle 1984b: 1103a14). Intellectual excellence requires time, education and experience, while moral excellence requires, as stated above, habit, which is defined in Aristotle’s *Metaphysics* as “a kind of activity of the haver and the had—something like action or movement.” Possessing a habit is a “disposition according to which that which is disposed is either well or ill disposed, either in itself or with reference to something else” (Aristotle 1984a: 1022b4–11; 1933). Though both intellectual and moral virtues are relevant here, the sense of virtue that we are specifically referring to above in relation to calling is moral virtue, which we will elaborate on below.

We are specifically concerned with moral virtues because they are dispositions of our emotions and rational faculties that help one respond correctly to practical situations (Hutchinson 1995: 206). For example, learning a particular craft well that is necessary for the practice of science. The activity of performing a certain task over time habituates one to learning how to perform the task or activity well. Aristotle’s example is, people “become builders by building and lyre-players by playing the lyre; so too we become just by doing just acts, temperate by doing temperate acts, brave by doing brave acts” (Aristotle 1984b; Hardie 1980: 104). For Thomas Aquinas, moral virtue is not to deprive the function of the emotions, but rather to “make them execute the commands of reason, by exercising their proper acts” (Aquinas 1948: I–II 59.4). Like Aristotle, Aquinas sees the “passions [as] a movement of the sensitive appetite when we imagine good or evil: in other words, passion is a movement of the irrational soul, when we think of good or evil” (Aquinas 1948). As skills are learned that relate to one’s natural gifting and calling, virtues, in the above sense, like prudence and what the ancients call, *techne* (art, craft) are developed.

In summary, callings relate to both the setting of ends and goals that we have identified as flourishing, and with the habitual processes that help achieve said flourishing. Having a calling allows for the habituation of virtue to come easier in some skills than in others. The doctrine of calling thus differs from many other ethics discussions on professions by emphasizing issues such as right assessment of ability, the responsibility for perpetual self-improvement and the duty towards community contribution through the use of talents.

### Why are Callings a Useful Way of Framing Science?

The idea of science as a calling instead of as a profession, art or practice thus introduces two important ideas that address the concerns outlined above. First, that the ability to function effectively as a scientist involves key dispositions that are by no means present in all individuals.^9^ Some individuals, it would seem, despite an interest in science are unsuited to the practice of scientific research, lacking the requisite talents. Conversely, some individuals may have an aptitude for science but no interest—or interest in other, competing callings. Moreover, even the most profound of callings requires a context that facilitates its enactment, emphasizing the importance of the research environment in the realization of science as a calling.

Second, the notion of science as a calling introduces the key issues of competing responsibilities and priorities that current discussions on science and responsible conduct find difficult to address. As scientists undoubtedly hold more than one calling, many of which compete regularly for pre-eminence, the doctrine of calling emphasizes the critical role that the broader community of scientists (and indeed more generally) plays in helping individuals negotiate these conflicts.

As noted, the means by which a calling is instantiated will be different from person to person. This is because, whereas the character of the virtues in a person are grounded in that person being of a certain kind, i.e. what she is essentially, calling is grounded in the conjunction of those virtues with the accidental intrinsic properties and relations of a person, i.e. what she is accidentally. Within a scientific context, this observation is also of key importance as it emphasizes the diverse roles within the practice of science—something not well elaborated within current responsibility discussions.

Within a number of individuals who have a calling to science as a practice (we will discuss below how to discern this), some may be more suited to teaching and mentorship roles, some to laboratory research, some to management and administration and so forth. Anyone within science will recognize the importance of ensuring that these diverse roles are represented within a laboratory, yet the *diversity of roles* within laboratories are often overlooked in responsibility discussions. Indeed, the majority of discussion—particularly on misconduct—remains at the level of individual laboratory research (Macrina 2007; National Academies 2009).

An engagement with the doctrine of calling thus offers a key insight into some important elements of scientific practice that are often overlooked by current science ethics discussions. In particular, it emphasizes not only the plethora of roles within the practice of science, but also the competing roles that individual scientists have to mediate within their daily activities. How, then, we ask, can science as a calling be realized, and what does it mean to “flourish” as a scientist when one is practicing one’s calling? We will now discuss the role that virtue and flourishing play within the calling to be a scientist.

Any such model would need to address key questions relating to the social nature of scientific research. First, how can the roles and responsibilities of the *scientific community* towards the individual be understood in terms of pedagogy and development? Second, what responsibilities does the individual hold in terms of doing “what is best for the community”? Finally, how can such responsibilities be enacted in daily research?

## Realising Callings

Realising science as a calling thus requires the identification of goals that facilitate flourishing. However, in realizing these goals, the doctrine of calling (in contrast to other ethical approaches) emphasizes not only the necessity for innate talent, but also the key role that the moral virtues play in habituating individuals to the calling. All of these are interesting in light of scientific practices, as discussed below.^10^

As noted, the means by which a calling is instantiated will be different from person to person. This is because the character of the virtues in a person are grounded in that person being of a certain kind. Within a scientific context, this observation is also of key importance as it emphasizes the diverse roles within the practice of science—something not well elaborated within current responsibility discussions.

Conversely, the doctrine of callings highlights the idea that *not all individuals should be scientists*. As a calling relates not only to the desire of an individual, but to the traits of a person—intellectual and moral virtues, but also talents—it is apparent that only some individuals will be suited to the pursuit of science. While current ethics discussions on science—particularly those relating to teaching through example—place considerable responsibility on mentors and peers to make sure that individuals are taught and supported in their daily activities nothing, conversely, is said about the responsibilities of the individual and the community for advising against the pursuit of science. In light of the considerable intellectual and tacit abilities necessary to successfully pursue a career in science, this omission is both worrying and odd.

What the idea of science as a calling highlights is not only the interest, dedication and discipline necessary to be a scientist, but also the innate talents necessary if one is to flourish as one. While, as mentioned above, one may flourish in many different roles as a scientist—as a teacher, researcher or administrator—it is nonetheless critical that one is in possession of certain key talents to flourish at all. What these talents are, of course, requires further examination.

What framing science as a calling does is to draw a distinction between good and excellent science practices. While it is possible to assume that even those without a calling towards science may become sufficiently accomplished to achieve competence within their daily activities, it is probable that they will not achieve excellence due to the absence of the talents necessary for science. Thus, some scientists may be perfectly competent, but lack the creativity, attention to detail or persistence necessary for greatness. This, of course, may not affect the quality of the individual research, but it influences the epistemic potential of science as a whole.

Recognizing that not all individuals who are interested in science are suited to science careers is an important topic of discussion for future ethics discourse—particularly discussions on responsibility. This is also a very valuable means of highlighting avenues through which the practice of science can be strengthened. The current global emphasis on STI and the corresponding push to get more students – particularly women—enrolled in STEM subjects is clearly an area in which these discussions are invaluable. Whether simply having *more* individuals trained as scientists (regardless of possessing a calling for scientist) are more valuable than fewer individuals truly called to scientific professions is something that the international community needs to discuss.

## Science as a Calling in Life Science Ethics

Framing science as a calling allows us to usefully discuss that which is well-known to the scientific community—that not all individuals are suited to careers in the lab. Some people, it seems, just don’t have the “hands” for benchwork, or could be proficient at benchwork but lack the joy necessary to find fulfillment in these activities. However, what the notion of science as a calling also allows us to do is to work through some of the ethical implications of callings and to identify key areas of responsibility amongst the scientific community that will enable the impasse described above to be addressed in a meaningful and robust manner.

Using calling to describe laboratory practice enables key issues to be raised in ethics discourse, namely who has responsibility for safeguarding science as a calling? In framing this question we use different loci of responsibility:

- Individuals have responsibility to the scientific communities to ensure that they are in the appropriate calling and that they have reconciled competing callings.
- Individual have responsibility to ensure that they are working in the calling that maximizes their talents and thus their contributions to their community.
- Scientific communities have a responsibility to ensure that they guide individuals in the recognition of their calling.
- Scientific communities have responsibilities to ensure that they foster environments in which individuals can realize—and flourish in—their calling.

Framing science as a calling represents a novel view of research that places the talents and dispositions of the individual and their relationship to the community at the center of flourishing practices. In the following sections we will examine how this reframing of scientific research assists in discussing the *relational responsibilities* highlighted above in a richer and more holistic manner. Importantly, we will discuss these responsibilities from two different perspectives: first, what responsibilities does the individual hold in terms of doing “what is best for the community” and how these are enacted in daily research. Second, the roles and responsibilities of the scientific community towards the individual in terms of pedagogy and development.

### Individual Responsibilities for Science as a Calling

As mentioned above, understanding science as a calling means that individuals practicing science have a number of key responsibilities. They must be able to ensure that—without self-deception—they are in the right calling. They must be critically self-aware of the talents and dispositions necessary to further the practice of the calling, and to place the good of the calling above their personal self-interests. They must also ensure that they are always striving to work in a way that maximizes their talents and thus their contributions to their community. Having a calling, after all, is a continuous state of being.

Returning to the Puritan literature on callings allows these responsibilities to be fleshed out in more detail. In particular, the literature emphasizes *self*-*reflection* as a key responsibility for any individual practicing a calling. Finding a calling is, in a sense, discovering one’s talents (what one can do) and one’s personality (what fits the person) (See also, Hardy 1990: 80; Veith 2002: 52–53). This, understandably, is much easier said than done, and literature—theology, psychology, sociology—abounds with examples of the difficulties of self-knowledge. Because of the difficulties of developing a comprehensive sense of self, it is possible for many to be deceived as to their vocational callings. This creates situations in which individuals may embrace—or desire—a profession that is unsuited to their talents or dispositions. Such is undoubtedly the case for science and scientists.

The problem here is an epistemic problem of how people can have confidence that what they are doing is actually their calling. In science, many individuals may believe that they have a calling to scientific research yet lack the tacit or intellectual dispositions necessary to flourish in their chosen profession. To refer back to the empirical work discussed above, some individuals just have “bad hands”.

In order to know that one has a certain gifting or calling, one must be justified in believing that one’s belief in possessing a calling is formed in a manner that is at least minimally reliable, that it has at least a minimally reliable source—self-reflection. This could be the reflection on the joy one receives from the activity, and on the progress of ‘getting better’ over time. An important aspect of scientific research that is missing from discussions on life science ethics—or indeed on most career advice in research—is the fundamentally important role that joy plays in successfully maintaining a scientific career. The notion of flourishing, so important to any calling, is patently absent from most discussions on scientific research and the importance of joy, wonder and awe in daily scientific research overlooked as peripheral or indulgent. By focusing on these necessary elements of flourishing it is possible that more scientists would be able to critically evaluate the fitness of scientific research as their calling.

By self-reflection, as well as by the increasing growth of joy and progress in doing a calling, people are at least minimally justified in believing that they are gifted in a certain activity. If someone were engaged in self-reflection, then they would recognize that they are naturally gifted at some activities and not at others. Alasdair MacIntyre (1999) notes that the virtues that are indispensably required for acquiring degrees of self-knowledge and preventing self-deception are “honesty, primary truthfulness about ourselves, both to ourselves and to others.”

Nonetheless, as philosopher Charles Taylor emphasises, our self-interpretations are based on, or are ‘constitutive’ of, our previous experience. Taylor puts it this way:

…because our insights into our own motivations and into what is important and of value are often limited by the shape of our experience, failure to understand a certain insight, or see the point of the moral advice proffered, is often taken as a judgment on the character of the person concerned (Taylor 1985: 38).^11^

Individuals thus need assistance in developing robust practices of self-reflection that assist them in understanding their calling *qua* science. Key topics to discuss could include how to reconcile priorities, find balance between roles, recognize competing responsibilities and prioritize non-science goals—such as home life and social relationships.

Over and above the importance of self-reflection as a key means of identifying real versus mistaken callings, it also has the potential to play important roles in strengthening the scientific practices of those for whom science really is a calling. In the literature above we highlighted that an individual in a calling becomes increasingly fitted to their role through habituation through practice. While this habituation is related to their natural gifts and ability, it is also a discipline that involves hard work and dedication. In this process continual self-reflection is a vital element. In developing excellence in science critical self-reflection would thus provide scientists with insight regarding their abilities. In particular, it would provide a tool through which to mediate between hubris and excessive self-criticism. Being able mediate between these two poles—both of which are all too common in narratives by scientists—may provide scientists with a valuable tool through which to avoid the mistakes and poor conduct that could arise from unrealized callings.

### Communal Responsibilities for Science as a Calling

Having individuals in the inappropriate professions has a number of epistemic and social consequences. Importantly, as observed by Perkins, individuals in mistaken callings can be hugely detrimental to these communities by disturbing the web of interlocking economic structures on which they depend for flourishing (Perkins 1603).^12^ Similarly, in science, the epistemic, economic and social implications of the scientific community retaining such individuals is huge as it potentially undermines the efficiency of scientific research as an endeavour.

Scientific research is essentially a communal practice—both epistemically and practically. To conduct scientific research is to continually do so in reference to others, and to recognize membership to institutional, national, and disciplinary and international communities of scientists. This “communality” of scientists is widely discussed from many perspectives—as a repository of scientific norms (Merton 1942), as an epistemic tool (such as promoted by the Sociology of Scientific Knowledge movement—see Shapin 1995), as a teaching tool (learning through example) and as justifications for responsibility to the public.

Communities thus have responsibilities to assist individuals with the discovery of their calling. There are many ways in which an individual can be deceived of their calling, and it is thus a responsibility of each member of the community to contribute external reflection and advice to individuals to assist with their development. This is not an *ad hoc* issue to be engaged with when convenient, but rather a fundamental aspect of being a scientist. The virtues necessary for self-examination but also in accountability to those with whom we participate in community, “those who have reason to look to us to help in meeting needs, by acknowledging to them our inadequacies and failures, wherever it is relevant to do so.” In order to be independent practical reasoners, we must concede to those who are expert co-workers, moral exemplars and friends. We have to rely on such people, from our close communities, friends and family members, for these necessary corrections (MacIntyre 1999: 95–97).

This is a departure from most ethics discussions that emphasize the role of the principle investigators (PIs) in discussions of responsibility and duty. Little—if anything—is said about the myriad of other roles and important loci of action within laboratories. In contrast to heading research projects, others may find meaning and value in supporting research (as technicians or more theoretically), by teaching and providing pastoral care to students and staff, or through the management and attention to detail necessary for laboratory managers. The recognition of these different roles within academic research is vital to the development of informed understandings of callings, and to the establishment of a stable and flourishing scientific community.

As is evident from codes of conduct, such as that of the American Chemical Society, it is widely recognized that the scientific community has responsibilities towards mediating the conduct of individual scientists. Individual scientists, by virtue of their membership, have important duties towards upholding the norms of science, safeguarding against harms,^13^ and training future generations of scientists.

While these duties are, of course, of particular importance for the perpetuation of scientific research, the discussion on community involvement in callings above brings another aspect to bear. In particular, it highlights the important responsibility of the scientific community in providing honest and truthful advice to scientists on finding or developing their callings. What is lacking from discussions on scientific communities is a rich and nuanced understanding of how each individual in these scientific communities has a duty not only to assist others to reflect on their calling to science, but to proactively assist them in their processes of self-reflection.

Whether scientific communities do indeed enter into such a commitment is obviously complicated. Diverse literature suggests that the necessary traditions of positive—yet critical—reflection and peer-guidance and pastoral care may be lacking from contemporary science. In particular, the increasing turn towards the commercialization of scientific research and the rising pressures of modern academia may actively thwart any attempts to foster such cultures.^14^

## Callings are All About Relationships …

What is evident from the discussion above is that understanding callings is all about understanding relationships—between the scientist and their discipline as well as their community. Scientists cannot engage in self- or communal-reflection without establishing effective relationships with their peers. What differs from other ethics discourse is that defining science as a calling makes the cultivation and nurturing of these relationships an ethical duty.

So, how do we understand these relationships that facilitate the identification of individual callings? In contemporary literature these relationships are rarely well-elaborated on. A review of current codes of conduct and ethics teaching highlight the following:

- That areas of social responsibility to peers are highly compartmentalized in discourse—mentoring, teaching from example, etc.—make it difficult to talk about *relational responsibilities* in general terms.
- That current discussions tend to overlook the key roles of pastoral care, friendship and support necessary for effective research environments.
- That current discussions on social responsibilities do not have space for discussions on talent, aptitude or differing abilities.

These issues have undoubtedly shaped ethics discussions on *relational responsibilities,* causing these responsibilities to be couched in specific terms. First and foremost, it is important to note that these discussions are often hierarchically focused and concentrate on the responsibilities of PIs and mentors—in contrast, the responsibilities of students and individuals not in teaching/leadership roles are not extensively discussed. Similarly, the expected outcomes of these responsibilities are positively-focused, in that the emphasis tends to be on supporting and developing new scientists.

Perhaps because of the focus on mentorship relationships, discussion on relational responsibilities also tends to focus on individual interactions and to provide rules or guidelines that define these relationships. A good example of this could be the student/mentor agreements that an increasing number of institutions are mandating. More general responsibilities to the scientific community at large tend to be very generally addressed mainly through codes of conduct or other aspirational statements. Because of this, discussions on relational responsibilities can be thought of as largely decontextualized from specific frames of reference.

While student/mentor agreements and other policies are undoubtedly influenced by related fields such as psychology, ethics discussions on inter-personal responsibilities are largely devoid of reference to these fields of study. Thus, while studies in psychology or theology could contribute to discussions on inter-personal relationships—particularly with regards to pastoral care—they do not feature in discussions on establishing and maintaining flourishing relationships. Similarly, discussions on work environments, social cultures and the establishment and perpetuation of ethical cultures (Bezuidenhout 2013) within laboratories is largely absent.

In contrast, understanding science as a calling highlights the critical *interconnectivity* and *co*-*dependency* of individual scientists and highlights the responsibilities that they have to each other. It would thus seem that a more care-oriented model is needed to infuse meaning into discussions on *relational responsibilities*. In particular, a model is needed that takes into consideration the responsibilities that an individual has to their peers, their community, and as a result of their membership to the community. Such a person-centric and contextual model may be valuable in breathing new life into discussions on responsibility, mentorship and “leading by example”.

Understanding science as a calling enables us to highlight the critical need to discuss the gap between proficiency and excellence. Thus, it enables us to talk concretely about the need to foster relationships in the laboratory that create spaces to talk about failure as well as success; establish environments that allow diversities of callings to be appropriately valued; recognize the multi-dimensional nature of peers and students and facilitate discussions that take their dispositions and extra-laboratory cultures into perspective; and take tacit ability as a talent instead of something that can always be cultivated through repetition.

The ability to talk about laboratory science as a calling is also vital to discussions with scientists who have not found their calling in the lab. Framing laboratory science as a calling enables frank discussions about alternative callings without the judgement and shame that often accompanies decisions to leave the laboratory.^15^ By placing alternative careers in science on suitable footing to laboratory research, this new framing will also assist in demystifying the specialized nature of science and its practitioners that have been described by many authors (Traweek 1992).

At the end of the day, framing laboratory science as a calling instead of a profession creates a space in which not being suited to this specific calling is not a failure, but rather a positive challenge to both the individual and to the scientific community to determine the calling to which they are suited. Recognizing that positive advice to those around you in the laboratory, when properly reasoned and informed, is an duty of all scientists will enable a more robust science as well as disseminating well-trained people into professions in which their understanding of science can be useful.

## Ethical Quandaries of Everyone as a Scientist

In addition to the value that a discourse on callings adds to science ethics and pedagogy, reanalyzing science through the lens of callings also draws attention to some key ethical quandaries that are perpetuated in the current research climate. First, as noted above, the increasing availability of science careers and funding for science education can result in proficient individuals engaged in science careers that are not their calling. This has epistemic consequences for science—as well as for the other careers that suffer by omission. Without hyperbole it is possible to suggest that individuals who are not called to science might lack the rigour and meticulousness necessary for exemplary science. In consequence, small mistakes and oversights and so forth could be perpetuated within scientific research. Without distinguishing between proficiency and excellence—in pedagogy as well as within ethics discussions—such instances will undoubtedly persist.

Second, the strict focus of academic pedagogy on laboratory research may cause individuals who have a calling within a related field of science (pedagogy, mentorship, management and so forth) to be excluded before reaching their potential. First and foremost, training high numbers of laboratory-proficient students with limited access to permanent laboratory-based research careers leads to a high drop-off amongst postdoctoral and early career researchers (Cyranoski et al. 2011; Powell 2015). There is an underlying assumption that “only the best survive” the challenges of postgraduate and postdoctoral research periods, and that candidates must be “hungry” for success if they are to survive. Candidates drop out for many reasons, including competing family commitments, concern about the unsocial hours of laboratory work, or a recognition (or a perception) that they are not as “good as their peers”.^16^ This huge drop-off of scientists after the postgraduate and postdoctoral stages also creates an unethical situation in that there is a waste of financial and pedagogical resources as individuals are trained in the intricacies of laboratory research without showing a propensity for it. This potentially pulls resources away from individuals who will be able to make successful careers in laboratory-based research. Without recognizing the heterogeneity of callings within—as well as outside—of science it is unlikely that such situations can be remedied.

While the focus of attention—and resources—on getting laboratory-proficient graduates detracts from other areas in which a basic undergraduate in science might be useful—teaching, journalism, and policy. While a number of universities in high-income countries (HICs) are starting to offer progressive programmes that combine the disciplines necessary for these professions, such programmes are still not the norm. Moreover, in low/middle-income countries (LMICs), where educational resources are most stretched, and the need for these alternative professions most pronounced, such programmes are largely absent.

Similarly, without having the recognition of science as a calling with which to discuss career decisions, many scientists leaving academic research feel a sense of failure and disappointment (in particular, see the posts on blogs.nature.com). This current situation creates an unhealthy situation in which individuals are placed under considerable strain—to their own and their discipline’s detriment. Even if they do not leave science, the strictly hierarchical structure of academic research often makes it difficult to discuss flourishing. The transitions between levels of seniority are marked by the assumption of changing duties and responsibilities, yet the transition points are poorly managed. Consequently, there is a potential to advance without self-examination and for individuals to continue in science careers without being suited to them. In addition, while many careers are open to individuals with science degrees outside of academia, the varying degrees of awareness of these options within the science community—as well as the difficulties of accessing them—can cloud processes of self-reflection with fear or pragmatic concerns.

To conclude, this paper has shown the manifold ways in which the notion of calling can be used as a tool to interrogate science. It has shown how current discourse structures in ethics, STS and science pedagogy are strengthened by the use of calling literature that highlights the importance of self- and communal-reflection. Moreover, the focus on joy and flourishing that are fundamental to the notion of calling refocuses attention on the overlooked distinction between proficiency and excellence—something that has significant implications for discussions on science. The use of calling literature in shaping understandings of science thus has both academic and practical implications—by influencing not only how we understand science, but how we teach, fund and oversee it.

## Footnotes

1
https://www.aip.org/fyi/2015/president-obama-stem-education (Accessed 14/01/2017).

2
https://www.aip.org/fyi/2015/president-obama-stem-education (Accessed 14/01/2017).

3 Two researchers, one of which was the author (LB) were embedded within two molecular biology laboratories at a private university within the USA to conduct ethnographic research. Starting in September 2016 these researchers will conduct their own science benchwork to better understand how science operates as a daily practice.

4 There are a number of ways in which post-reformation thinkers conceived of how callings manifest themselves in the world.  In this paper we confine ourselves to the work of Perkins, but would direct interested readers to the work of John Calvin and Martin Luther.

5 We need to highlight in this paper our limited use of the term calling. In this paper we are strictly using the term calling to refer to the workplace, and specifically laboratory science. As has been shown above, calling can extend into all aspects of life including those activates and roles that we engage with outside our workplace. In this paper we are only discussing the calling to a certain vocation within one’s socio-political context.

6 This refers to the ‘supernatural’ and ultimate flourishing that only takes place in the next life. The achievement of this end is a gift and in not able to be achieved without faith.

7 For literature in the theological tradition that supports the claim of of earthly flourishing see, (Augustine 1984: XIX, 20; Aquinas 1948: I II, 4, 5; 5, 5, 7; 62, 1; 63, 3; Milbank 2005: 60; de Lubac 1998; For more on this within reformation theology see Warne 2016).

8 We will here not address the debate of the unity of the virtues or the virtues relation to nature of metaphysics.

9 It is also important to differentiate the focus of our argument—namely, that individuals may be called to the profession of scientific research in a similar manner to those called to teaching, medicine or nursing. We do not in this paper examine the nature of science itself.

10 For more on the relationship between scientific practice and virtue see (Stapleford and Hicks 2016).

11 The fuller context of this discussion is in first and second order evaluations against utilitarian decision-making procedures, but this small bit of his larger argument shines some light on our current discussion.

12 Indeed, this may be taken as the case regardless of the external goods motivating scientists in their calling—for instance hypothesis testing, achieving ends of a practical and//or commercial nature. The achievement of any of these external goods relies on an robust community of scientists.

13 Such as the dual-use of scientific knowledge, where beneficial research may be misapplied for harmful purposes by a third party. For a good introduction see Miller and Selgelid (2008).

14 This has been taken up in the media, examples of which can be found here: http://www.economist.com/news/leaders/21588069-scientific-research-has-changed-world-now-it-needs-change-itself-how-science-goes-wrong or here http://www.economist.com/news/briefing/21588057-scientists-think-science-self-correcting-alarming-degree-it-not-trouble (accessed 8 June 2016).

15 A good place to find these discussions are in the numerous blogs by postdoctoral scientists and researchers. See, for example, http://blog.devicerandom.org/2011/02/18/getting-a-life/ and http://www.benchfly.com/blog/lessons-from-a-recovering-postdoc/ (Accessed 16/01/2017).

16 See footnote 14.
